# Cyclin-dependent kinase 5 promotes the stability of corneal epithelial cell junctions

**Published:** 2013-02-07

**Authors:** Parthasarathy Arpitha, Chun Y. Gao, Brajendra K. Tripathi, Senthil Saravanamuthu, Peggy Zelenka

**Affiliations:** 1 National Eye Institute, NIH, Building 5635, Room 1S-02, Fishers Lane, Rockville, MD; 2 National Eye Institute, NIH, Building 6, Bethesda, MD

## Abstract

**Purpose:**

Although cyclin-dependent kinase 5 (Cdk5) inhibits the formation of junctions containing N-cadherin, the effect of Cdk5 on junctions containing E-cadherin is less clear. The present study investigates the functional significance of Cdk5 in forming and maintaining cell–cell stability in corneal epithelial cells.

**Methods:**

A Cdk5-deficient human corneal limbal epithelial cell line was generated by lentiviral transduction of small hairpin RNA specific for Cdk5 (shCdk5-HCLE cells). A blasticidin-inducible vector for expression of Cdk5-specific short hairpin RNA (ShCdk5) was generated by recombination and packaged into non-replicative lentiviral particles for transduction of human corneal limbal epithelial (HCLE) cells. Blasticidin-resistant cells were isolated for analysis. Cell aggregations were performed using HCLE, Cdk5 inhibitor olomoucine, ShCdk5, and MDA-MB 231 cells in the presence and absence of calcium, and particle size was measured using image analysis software. Relative protein concentrations were measured with immunoblotting and quantitative densitometry. Total internal reflection fluorescence (TIRF) microscopy was performed on cells transfected with green fluorescent protein (GFP)-E-cadherin or GFP-p120, and internalization of boundary-localized proteins was analyzed with particle tracking software. The stability of surface-exposed proteins was determined by measuring the recovery of biotin-labeled proteins with affinity chromatography. Rho and Rac activity was measured with affinity chromatography and immunoblotting.

**Results:**

Examining the effect of Cdk5 on E-cadherin containing epithelial cell–cell adhesions using a corneal epithelial cell line (HCLE), we found that Cdk5 and Cdk5 (pY15) coimmunoprecipitate with E-cadherin and Cdk5 (pY15) colocalizes with E-cadherin at cell–cell junctions. Inhibiting Cdk5 activity in HCLE or suppressing Cdk5 expression in a stable HCLE-derived cell line (ShHCLE) decreased calcium-dependent cell adhesion, promoted the cytoplasmic localization of E-cadherin, and accelerated the loss of surface-biotinylated E-cadherin. TIRF microscopy of GFP-E-cadherin in transfected HCLE cells showed an actively internalized sub-population of E-cadherin, which was not bound to p120 as it was trafficked away from the cell–cell boundary. This population increased in the absence of Cdk5 activity, suggesting that Cdk5 inhibition promotes dissociation of p120/E-cadherin junctional complexes. These effects of Cdk5 inhibition or suppression were accompanied by decreased Rac activity, increased Rho activity, and enhanced binding of E-cadherin to the Rac effector Ras GTPase-activating-like protein (IQGAP1). Cdk5 inhibition also reduced adhesion in a cadherin-deficient cell line (MDA-MB-231) expressing exogenous E-cadherin, although Cdk5 inhibition promoted adhesion when these cells were transfected with N-cadherin, as previous studies of Cdk5 and N-cadherin predicted. Moreover, Cdk5 inhibition induced N-cadherin expression and formation of N-cadherin/p120 complexes in HCLE cells.

**Conclusions:**

These results indicate that loss of Cdk5 activity destabilizes junctional complexes containing E-cadherin, leading to internalization of E-cadherin and upregulation of N-cadherin. Thus, Cdk5 activity promotes stability of E-cadherin-based cell–cell junctions and inhibits the E-cadherin-to-N-cadherin switch typical of epithelial–mesenchymal transitions.

## Introduction

Cdk5 is an atypical member of the cyclin-dependent kinase (Cdk) family, which has no known role in cell cycle regulation [1]. Cdk5 is primarily expressed in central nervous system neurons, but lower levels of expression and activity are present in a wide variety of tissues, including the corneal epithelium [2,3]. Cdk5 is catalytically activated by dimerization with a regulatory subunit, p35 or p39 [4,5], and its basal activity may be further enhanced by phosphorylation at Y15 [6,7]. In migrating corneal epithelial cell sheets, we observed that Cdk5 (pY15) is predominantly localized along the leading edge, and phosphorylation of Cdk5 was Src dependent [2].

Cadherin-based cell–cell junctions, or adherens junctions, provide the major force for cell–cell adhesion in epithelial tissues and are critical for maintaining the integrity of the epithelial cell sheet. In most epithelial tissues, the type I membrane protein, E-cadherin, is principally responsible for forming adherens junctions. The E-cadherin ectodomain forms Ca^2+^-dependent homodimers with the ectodomain of E-cadherin on a neighboring cell, while the cytoplasmic domain associates with intracellular proteins, including p120, β-catenin binding to IQGAP1, and α-catenin, which stabilize the junction and link it to the actin cytoskeleton. Cadherin signaling at the membrane is also reported to be regulated by the GTPases, as activation of Rac antagonizes the binding of IQGAP1 to the junctional complex and suppression of Rho activity participates in promoting cell–cell interactions [8,9]. Cadherin-mediated cell–cell adhesion is controlled by tyrosine phosphorylation of p120, a Src substrate and a component of the junctional complex that modulates cadherin membrane trafficking and degradation [10]. Phosphorylation of p120 catenin by Src kinase triggers the dissociation [11]. The critical decision point for internalized E-cadherin is marked by Src-dependent phosphorylation, which targets E-cadherin for ubiquitination [12] and lysosomal degradation [5]. The cadherin-catenin clusters are also known to be regulated by the Rho kinase, which also functions either upstream or downstream of p120 in cell–cell adhesion [10]. Since the absence of Cdk5 activity and expression leads to a partial loss of cell–cell adhesion, the present study was undertaken to understand the mechanism of regulation of Cdk5 at the cadherin-based cell–cell junctions.

In a previous study, we observed that inhibition of the proline-directed kinase, Cdk5, tends to disrupt cell–cell adhesion in migrating corneal epithelial cell sheets during wound healing [2]. The adherence junctions of the corneal epithelium between the cells and the matrix confer a strong integral base for supporting normal vision. The mechanism of wound repair and during normal epithelial self-renewal enables the weakening of the bonds between the cells allowing proper migration of the epithelial cells [13]. Since studies from many laboratories have demonstrated that Rho-family GTPases and Src couple the regulation of cell–cell and cell-matrix adhesion during migration [14-19], we expected that inhibiting Cdk5 might reduce cell–cell adhesions as well. Further, we reported earlier that Cdk5 inhibition promotes migration of epithelial cells by reducing the cell to matrix interactions and have shown that such inhibition is mediated by Rho-GTPase and Src [20-22]. Although earlier reports from several laboratories and from our previous findings in lens epithelial cells expressing N-cadherin and in vivo cornea E-cadherin indicated the importance of Cdk5 in migration, the mechanism of regulation of Cdk5 at cell–cell adhesions has been less explored. Therefore, the present experiments were undertaken to clarify the role of Cdk5 in regulating E-cadherin dependent junctions in corneal epithelial cells.

## Methods

### Generation of human corneal limbal epithelial cells lacking the expression of cyclin-dependent kinase 5

The immortalized corneal and limbal epithelial cells (HCLE) were cultured as described [23]. Briefly, cells were grown in a medium nutritionally optimized for growth of keratinocytes: keratinocyte serum-free medium (Gibco-Invitrogen Corp., Rockville, MD), supplemented with 25 μg/ml bovine pituitary extract, 0.2 ng/ml epidermal growth factor, 0.4 mM CaCl_2_, penicillin (100 units/ml), and streptomycin (100 μg/ml) at 37 °C in a humidified atmosphere of 95% air and 5% CO_2_. About 15 μM olomoucine (Calbiochem, San Diego, CA) was used in the HCLE medium to inhibit kinase activity.

A blasticidin-inducible vector for expression of a Cdk5-specific short hairpin RNA (ShCdk5) was constructed by recombination and packaged into non-replicative lentiviral particles and used for transduction on to the HCLE cells. The Cdk5-pLentivirus titer was more than 3.5×10^−4^ TU/ml. Blasticidin-resistant (2.5 μg/ml) Cdk5-deficient cells were selected, and the CDK5-deficient cell line (ShHCLE) was stored for further usage.

### Immunoblotting and immunoprecipitation

Immunoblotting and immunoprecipitation were performed as previously described [24]. Cellular proteins were lysed and extracted in radioimmunoprecipitation assay (RIPA) buffer (1% NP-40, 50 mM Tris-HCl at pH 7.4, 0.5% Na-deoxycholate, 150 mM NaCl, and 0.1% sodium dodecyl sulfate) containing one cOmplete-Mini protease inhibitor cocktail tablet per 10 ml and tyrosine and serine/threonine protein phosphatase inhibitor cocktail (Upstate Technology, Lake Placid, NY). Twenty-five μg of total cell extract was used for immunoblotting and 200 μg for immunoprecipitation. Immunoprecipitated proteins were collected on magnetic beads (Invitrogen), eluted with loading buffer, and immunoblotted. Immunoreactive bands were detected with enhanced chemiluminescence (ECL-Plus; GE Healthcare, Piscataway, NJ). Rho activity was detected using Rhotekin beads (Millipore, Billerica, MA).

### Antibodies

The following antibodies were obtained from Santa Cruz Biotechnology (Santa Cruz, CA): anti-Cdk5 mouse monoclonal (DC-17; sc-249), anti-Cdk5 rabbit polyclonal (C-8, sc-173), anti-pY15-Cdk5 (sc-12918), and anti-p35 rabbit polyclonal (C-19). Anti-E-cadherin (cat. no. 610,182), p120 catenin (cat. no. 610,133), IQGAP1 (cat. no. 610,611), isotype control mouse immunoglobulin G subclass 1 (IgG_1_), and IgG_2a_ were from BD Transduction Laboratories (Lexington, KY). Anti-M6PR, anti-LAMP2, and horseradish peroxidase-linked secondary antibodies were from GE Healthcare. Secondary antibodies tagged with Alexa Fluor 350, Alexa Fluor 488, or Alexa Fluor 568, and Alexa Fluor 568 phalloidin conjugates were from Molecular Probes (Carlsbad, CA). The Rho activation kit was from Millipore.

### Immunofluorescence

HCLE cells grown to 95% confluence on glass chamber slides (Lab-Tek, Scotts Valley, CA) were rinsed with phosphate buffered saline (PBS) with calcium and magnesium (Cellgro, Manassas, VA), fixed in 4% paraformaldehyde for 10 min, and permeabilized in 0.25% Triton-X100 in PBS for 3 min. Following several washes in PBS and blocking in 5% normal goat serum in PBS, slides were incubated with primary antibodies and corresponding isotype IgG for control slides for 1 h at room temperature, followed by incubating with secondary antibody conjugated to the fluorochrome.

After extensive washing in PBS, samples were incubated with the appropriate fluorescence-tagged secondary antibodies, followed by overnight washing in PBS. Slides were mounted in antifade reagent (Molecular Probes) and viewed with epifluorescence with a Zeiss AxioPlan 2 ((Thornwood, NY) microscope equipped with Zeiss AxioVision CCD camera.

### Transfections

HCLE cells at 60% confluence were transfected with either pBS/U6-si Cdk5 [25] and pEGFP C-1 (Clontech, Mountain View, CA), E-cadherin-GFP or P120-GFP using FuGENE 6 (Roche diagnostics, Indianapolis, IN). At specified times after transfection, cells were either fixed in glass chamber slides for immunofluorescence, or proteins were extracted for analysis by immunoprecipitation and immunoblotting.

### Confocal and total internal reflection fluorescence microscopy

A Leica TCS-SP2 laser scanning confocal microscope (Leica Microsystems) was used for colocalization of E-cadherin (imaged with Alexa 488-coupled goat antimouse IgG, excitation, 488 nm) and late endosome/lysosome marker M6PR (imaged with Alexa 568-coupled goat antirabbit IgG, excitation, 568 nm). HCLE cells treated with/without olomoucine and ShCDK5 were transfected with E-cadherin-pEGFP or p120-pEGFP tracked for the endocytotic movement of the junctional complexes using total internal reflection fluorescence (TIRF) microscopy. Several fields with numerous cells and about 300 (three separate experiments, n=3) particle vesicles containing E-cadherin or p120 were analyzed for the internalization. TIRF analysis was performed for only the particles that had movement, and these particles were chosen arbitrarily all along the cell borders. Images were recorded in the TIRF mode using the 488 nm laser scanner at 20 frames per second for a total of 48 s. The sensitivity cutoff for the TIRF analysis was set as the distance of a particle moving at a speed of 3.0 and tortuosity less than 1.5. Particle tracking was performed using the Zeiss Axiovision software, and the distance and tortuosity of the particles were recorded. Particles at a distance and tortuosity less than 1.5 were considered slow-moving particles while the fast-moving particles that traveled at a distance of 1.5 and tortuosity greater than 3 were the endocytosed junctional complexes.

### Cell aggregation assay

To functionally study the regulation of CDK5 on the formation or disruption of cell–cell junctions, a cell aggregation assay was performed as described earlier [22]. Briefly, the confluent HCLE [23] and rabbit lens epithelial cell line N/N1003A [22] and E-cadherin-deficient human epithelial breast adenocarcinoma cell line MDA-MB-231 [26] cells were treated with olomoucine (15 μM). These cells with or without olomoucine and ShCDK5 were rinsed in PBS free from Ca^2+/^Mg^2+^ and incubated with TrypLE (Gibco) containing 1 mM CaCl_2_ for 45 min with mild agitation to obtain a single cell suspension of cells. The cells were washed, and 1×10^6^ cells were allowed to form aggregates at 120 revolutions per minute for 60 min in the presence or absence of Ca^2+^. The cellular aggregates were fixed in 4% paraformaldehyde and cytospin on glass slides and stained with Giemsa. The area of the aggregate size was measured using the Zeiss AxioVision software.

### Glutathione-S-transferase pull-down assay

Glutathione-S-transferase (GST) pull-down assay was used to confirm the interaction of Cdk5 and E-cadherin with purified GST-Cdk5 to pull down E-cadherin from the HCLE cell lysate. One mg of whole cell lysates (lysed in 1% Triton X-100, 150 mM NaCl, 2 mM MgCl_2_, 2 mM CaCl_2_, 20 mM Tris-HCl, pH 7.4 containing protease inhibitors) was incubated with either 5 μg of GST-Cdk5 or GST only as control, and 0.5 ml of Glutathione-Sepharose 4B beads (50% slurry, Amersham Biosciences, Piscataway, NJ) in 5 ml of lysis buffer. The mixture was then incubated overnight at 4 °C with agitation. Beads were washed three times by suspending in 1 ml of wash buffer (PBS supplemented with 0.1% Triton X-100 and 10 mM β-mercaptoethanol) and centrifuging at 750 ×*g* for 5 min. The resuspended beads were eluted with 2X sodium dodecyl sulfate (SDS) sample buffer, heated, and analyzed with sodium dodecyl sulfate–polyacrylamide gel electrophoresis.

### Cell surface biotinylation

Confluent HCLE cells grown on six well plates were incubated with 1.5 mg/ml sulfosuccinimidyl 2-(biotinamido)-ethyl-dithiopropionate (sulfo-NHS-SS-biotin; Pierce Biotechnology, Inc, Rockford, IL) for 30 min followed by washing with sulfo-NHS-SS-biotin blocking reagent (50 mM NH_4_Cl in PBS containing 1 mM MgCl_2_ and 0.1 mM CaCl_2_) to quench free sulfo-NHS-SS-biotin and several additional washes in PBS. Cells were then incubated in the presence or absence of olomoucine (15 μM) in normal growth media at 37 °C for 4.5 h. To obtain the total biotinylated proteins remaining at the end of the incubation, cells were directly lysed after several washes with PBS. To obtain the pool of E-cadherin that was internalized by endocytosis, the cells were stripped of surface label with glutathione before lysis, by incubating in two 20 min washes of glutathione solution (60 mM glutathione, 0.83 mM NaCl, with 0.83 mM NaOH and 1% bovine serum albumin added before use) at 4 °C [27]. The remaining biotinylated proteins, sequestered inside cells by endocytosis, were protected from glutathione stripping. Cells were then harvested, sonicated, and lysed in 300 µl of RIPA buffer (20 mM Tris-HCl, pH 7.4, with 150 mM NaCl, 0.1% SDS, 1% Triton X-100, 1% Na-deoxycholate, and 5 mM EDTA) supplemented with protease inhibitors. The RIPA-soluble supernatant was incubated with streptavidin beads (Sigma Chemical, St. Louis, MO) to collect bound, biotinylated proteins, which were then eluted, analyzed with sodium dodecyl sulfate–polyacrylamide gel electrophoresis, and immunoblotted for E-cadherin. All quantitation was done with densitometry and normalized by immunoblotting for tubulin in the flow-through from the streptavidin affinity purification column.

### Image analysis

Images were analyzed using Image-Pro Plus (Media Cybernetics, Silver Spring, MD) unless otherwise specified. E-cadherin border localization (defined as the ratio of border intensity/total intensity) was determined [28]. Adjacent pairs of transfected or non-transfected cells in the same culture were randomly chosen for analysis. Transfected cells were identified by the fluorescence of a cotransfected GFP plasmid. The region of the cell–cell contacts containing E-cadherin accumulation were selected automatically by the software, and the average pixel intensity within the selected region was recorded. The average total E-cadherin intensity was measured by selecting the entire area covering two adjacent cells. Background intensity, determined by selecting an empty area, was subtracted from the border intensity and the total intensity before the border intensity/total intensity ratio was calculated.

Leica colocalization software was used to analyze and quantify colocalization of E-cadherin with M6PR and LAMP2 immunofluorescence. The total immunofluorescence was plotted as a cytofluorogram, and a rectangular region containing pixels with high fluorescence (>100 out of 256 maximum) in both channels was taken to represent colocalization. The percentage of the total E-cadherin immunofluorescence within the colocalized region was determined for images of eight different fields captured at 100× magnification, and the results were averaged. Single immunostaining for E-cadherin and p120 were quantified using ImagePro Plus software.

### Statistical analysis

All statistical analysis was performed using SigmaStat 2.0 (Systat Software Inc., San Jose, CA). Statistical significance was determined with the Student *t* test or rank-sum test, and the results were plotted as averages±standard error of the mean (SEM).

## Results

### Cyclin-dependent kinase 5 interacts with E-cadherin at cell–cell junctions

**Figure 1:**
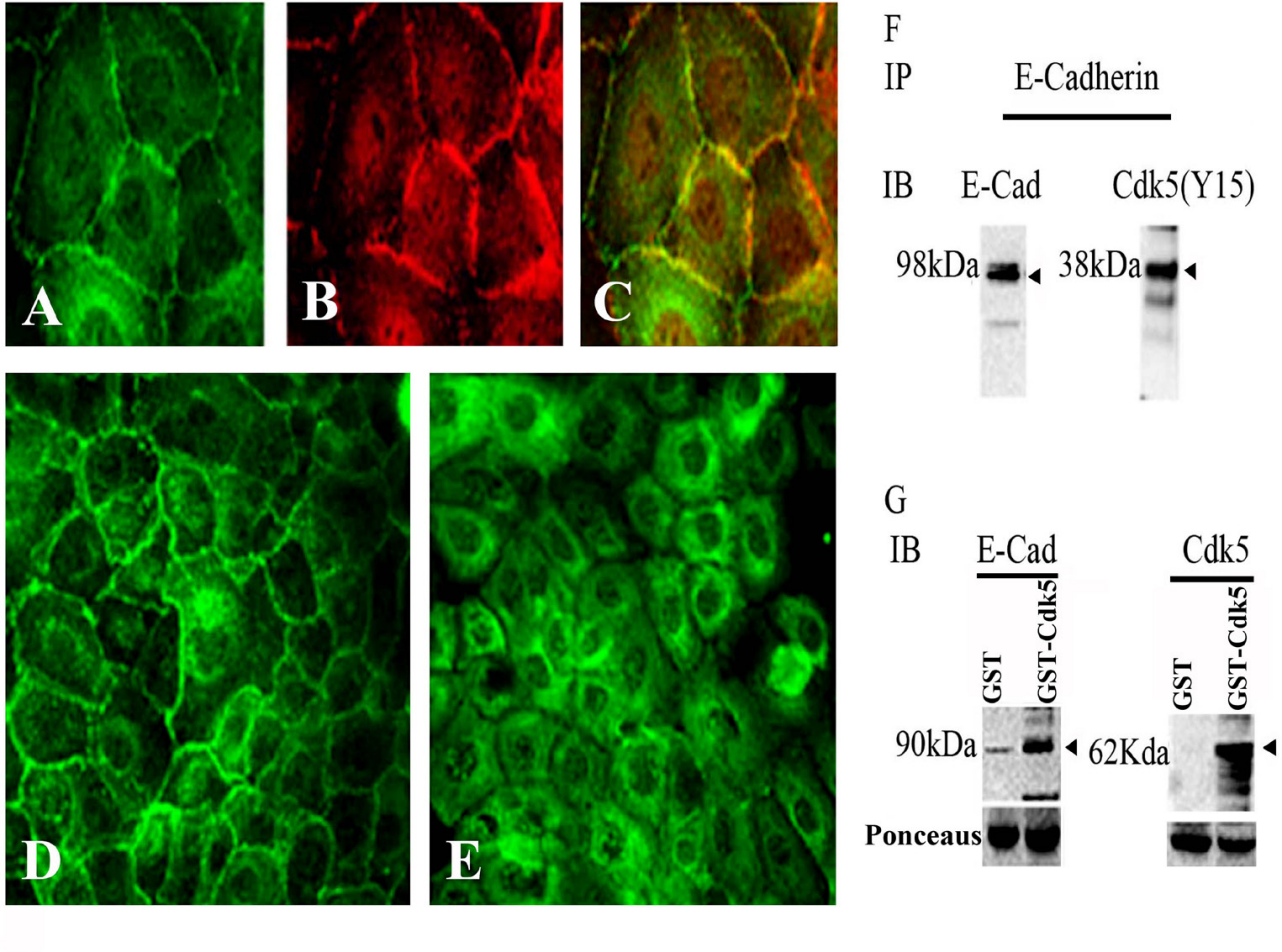
Co-localization of E-cadherin and cyclin dependent kinase5 (Cdk5) in the cell–cell borders. Human corneal limbal epithelial (HCLE) cells were grown on chamber slides, fixed, and immunostained stained with pY15 (green; **A**) and E-cadherin (red; **B**). Cyclin-dependent kinase 5 (Cdk5) and E-cadherin were localized to the cell–cell boundaries, and the overlay image demonstrates the colocalization (**C**) of E-cadherin and phosphorylated (pY15) CDK5. Confluent cultures of HCLE showing CDK5 border localization (**D**) confluent HCLE cultures when treated with olomoucine show a shift in the localization of pY15 CDK5 from the cell borders to the interior (**E**). E-cadherin and CDK5 form a part of the same protein complex (**F**, **G**) E-cadherin coimmuneprecipitates with CDK5 (38 kDa; F). Affinity chromatography for HCLE cell lysates were pulled down for glutathione-S-transferase-cyclin-dependent kinase 5 (GST-CDK5) and that glutathione beads bound to CDK5 formed a complex with E-cadherin (**G**). (**A**, **B**, **C**) 100X images and (**D**, **E**) 40X images.

To explore the possible role of Cdk5 at epithelial cell junctions, we first investigated the subcellular localization of Cdk5 (pY15), an activated, phosphorylated form of Cdk5. The results demonstrated that Cdk5 (pY15) was concentrated at cell–cell junctions in the HCLE cells, where it colocalized with E-cadherin (Figure 1A–C). Similar staining was seen using a Cdk5 antibody, although background staining was higher than for Cdk5 (pY15; not shown). Interestingly, the border localization of Cdk5 (pY15) was entirely lost when CDK5 activity was inhibited with olomoucine, implying that Cdk5 kinase activity is required to maintain this localization (Figure 1D,E). To determine whether the junctional localization of Cdk5 (pY15) might reflect a physical association of Cdk5 with the junctional complex, we next tested whether Cdk5 (pY15) forms a protein complex containing E-cadherin. Immunoprecipitating E-cadherin and immunoblotting for Cdk5 (pY15) confirmed that Cdk5 is part of an intracellular complex that includes E-cadherin. As an additional test of the ability of Cdk5 to interact with E-cadherin, we performed affinity chromatography with bacterially produced GST and GST-Cdk5 on glutathione-linked agarose beads (“GST pull-down” experiments). Proteins that eluted from the beads with glutathione were immunoblotted with antibodies to Cdk5 and E-cadherin. A strong E-cadherin band appeared in the GST-Cdk5 elute, but not in the GST control (Figure 1F,G), confirming that Cdk5 and E-cadherin are part of an intracellular protein complex.

**Figure 2:**
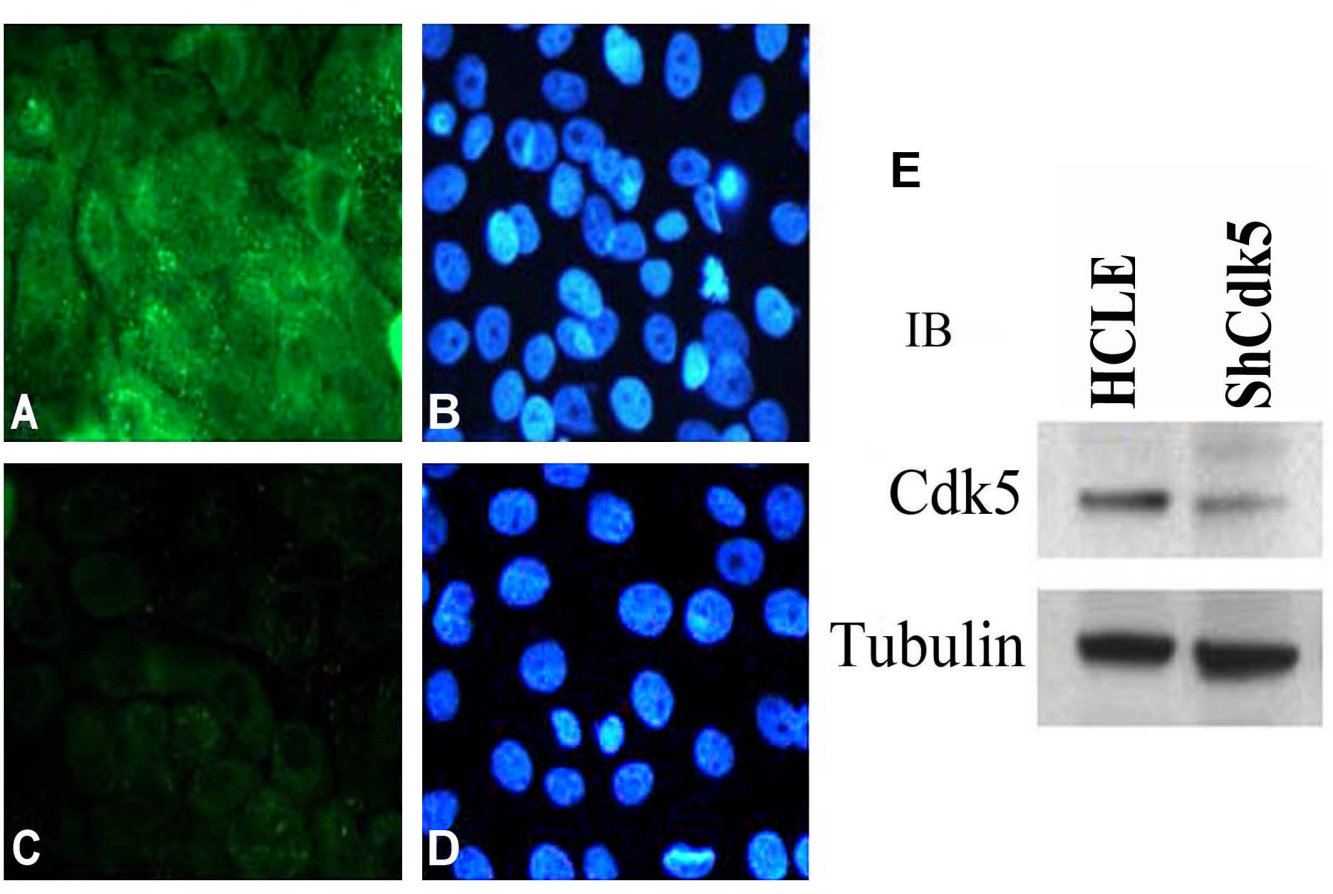
Human corneal limbal epithelial cell line suppressed for cyclin-dependent kinase 5 expression. Immunofluorescence image showing staining for cyclin-dependent kinase 5 (CDK5) (green; **A**) in human corneal limbal epithelial (HCLE) cells and note the absence of Cdk5 in the ShCDK5 cells (**C**). Nuclear counterstaining 4', 6-diamidino-2-phenylindole (blue) for images in **A** and **C** (**B**, **D**). Lysates from these cells showing significant reduction in the expression levels of CDK5 in the plentiviral transduced HCLE (ShCDK5) cells (**E**). 100X images.

**Figure 3:**
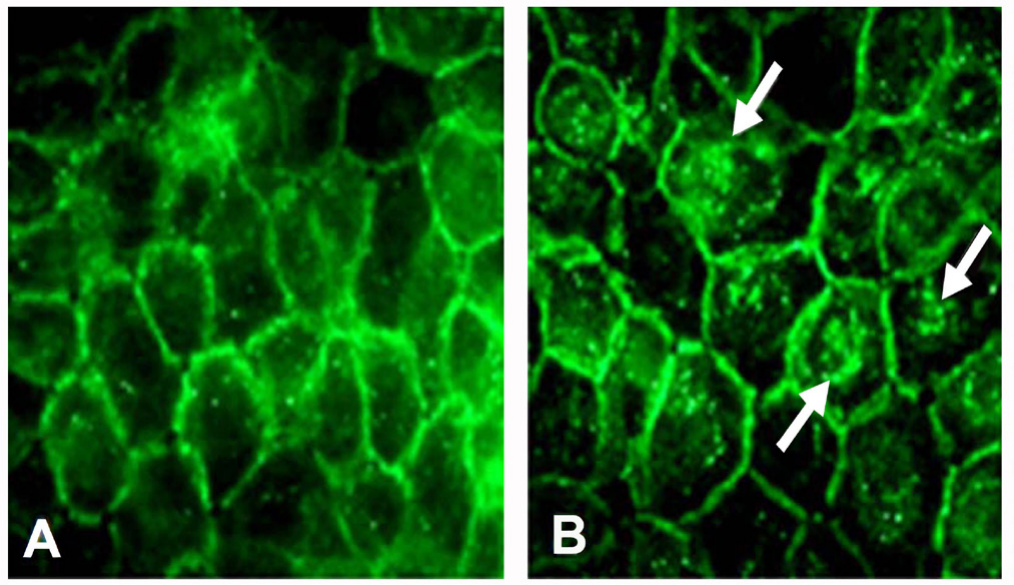
Cell–cell adhesions in the ShCDK5 cells. Confluent human corneal limbal epithelial (HCLE) and ShCDK5 cells immunostained with E-cadherin. The HCLE cells show border localization of E-cadherin (**A**). ShCDK5 increases the border localization to the cell interior marked by punctuate staining (arrows) within the cells. The ratio of the border to cell interior was significantly reduced in the ShCDK5 E-cadherin-labeled cells (0.347±0.145) when compared to the HCLE (0.535±0.142) 100X images.

Since these findings suggested that Cdk5 may have a functional role at epithelial cell–cell junctions, we next generated a Cdk5-deficient cell line to provide an additional experimental tool for examining Cdk5 function. Immunofluorescence (Figure 2A,B) and immunoblot (Figure 2E) for Cdk5 showed more than 90% suppression of endogenous CDK5 (Figure 2C), which remained suppressed for Cdk5 after multiple passages. We next used this line to test whether Cdk5 suppression affects the border localization of E-cadherin. To provide a quantitative measure of E-cadherin border localization, we determined the ratio of E-cadherin immunofluorescence at the border between two adjacent cells to the total E-cadherin immunofluorescence within those cells, as previously described [28]. This method indicated that approximately 53% (border/total=0.535±0.142) of the total E-cadherin immunofluorescence was localized at cell–cell junctions in untransduced HCLE cells. In contrast, only 35% (border/total=0.347±0.145) was localized at junctions in the ShHCLE line, indicating that suppression of Cdk5 expression with ShRNA significantly reduced the border localization of E-cadherin (p≤0.005; Figure 3). Similar measurements of p120 localization showed no significant difference between the HCLE (border/total=0.516±0.0291) and ShHCLE cells (border/total=0.576±0.027). These findings suggest that loss of Cdk5 may lead to dissociation of the E-cadherin/p120 complexes and internalization of E-cadherin.

**Figure 4:**
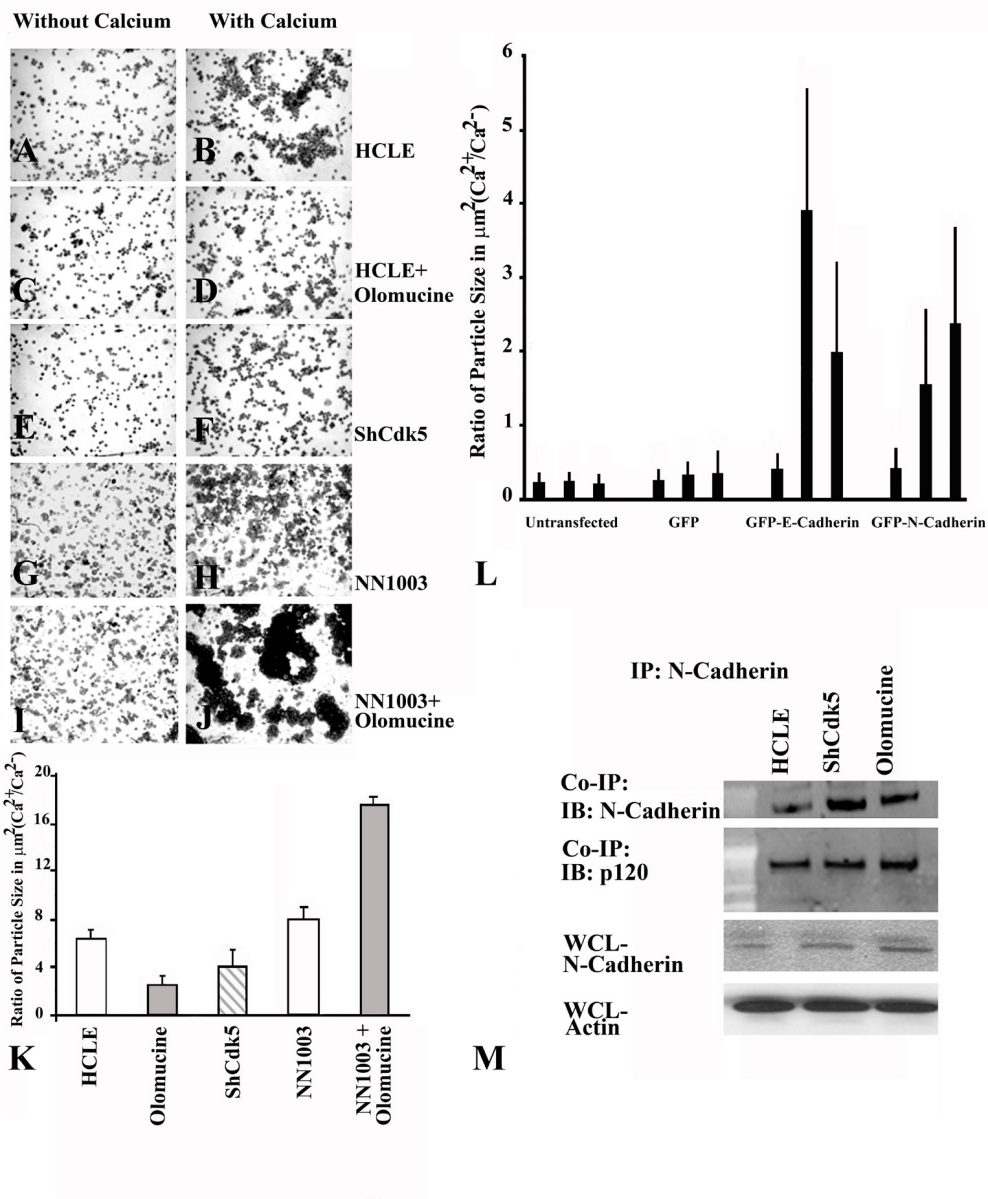
Cell-cell junction formation in the ShCDK5 human corneal limbal epithelial cells. Human corneal limbal epithelial (HCLE) cells (**A**, **B**), in the absence of cyclin-dependent kinase 5 (CDK5) with olomoucine (**C**, **D**) or ShCDK5 (**E**, **F**) and lens epithelial cells (NN1003; **G**, **H**) were treated with olomoucine (**I, J**). The cells were allowed to form cell–cell adhesions in the presence or absence of calcium for 1 h at 37 °C, and particle aggregates were cytospin on to glass slides to measure the size of the aggregates. Calcium-dependent E-cadherin junctions were formed in the presence of CDK5 (**B**, **K**) in the HCLE cells in contrast to N-cadherin expressing lens epithelial cells that form larger aggregates in the absence of CDK5 (**J**, **K**). (**A**–**J**) 20X images. E-cadherin and N-cadherin induction during cell–cell junction formation in the MDA-MB-231 cells (**L**). Green fluorescent protein (GFP)-E-cadherin and GFP-N-cadherin transfected cells were allowed to form cell–cell aggregates in the absence of calcium (lane 1), presence (lane 2) and with olomoucine (lane 3). Cells containing E-cadherin form more cell–cell aggregates (group 3) while the N-cadherin (group 4) aggregates increases in the presence of olomoucine. N-cadherin induction in the ShCDK5 and olomoucine HCLE (**M**) CDK5 inhibition leading to degradation of E-cadherin leads to induction of N-cadherin. P120 coimmuneprecipitates with N-cadherin to stabilize the junctional complex in the ShCDK5 and olomoucine-treated HCLE. IP=immunoprecipitation; WCl=whole cell lysate.

Since these results indicated that Cdk5 activity promotes junctional localization of E-cadherin, we expected Cdk5 activity would also affect calcium-dependent junction formation. To test this possibility, we performed an aggregation assay, comparing junction formation in HCLE cells, HCLE cells treated with the Cdk5 inhibitor, olomoucine, and Cdk5-deficient ShHCLE cells (Figure 4). The results showed that aggregates formed in the presence of olomoucine (Figure 4C,D) as well as those formed by ShHCLE cells (Figure 4 E,F) were significantly (p≤0.001) smaller than those formed by HCLE cells (Figure 4 A,B), indicating that Cdk5 activity promotes calcium-dependent junction formation. Since this result differed from results we had previously reported [22] using the lens epithelial cell line N/N1003A, which expresses primarily N-cadherin, we repeated the aggregation assay on the N/N1003A cells to ensure that the assay conditions were identical. The results confirmed our previous finding: Inhibiting Cdk5 activity increases the size of the aggregates formed by N/N1003A cells (Figure 4I–J). The quantitative results of the aggregation assays are summarized in Figure 4K. These results indicate that loss of Cdk5 activity inhibits calcium-dependent junction formation in HCLE cells, but not in N/N1003A cells, possibly due to the difference in the cadherin.

### Differential effect of cyclin-dependent kinase 5 on E-cadherin and N-cadherin

To test if the effect of Cdk5 inhibition depended on the type of cadherin expressed, MDA-MB 231, a cell line devoid of cadherin expression, was transfected with GFP-E-cadherin or GFP-N- cadherin. The aggregation assay performed suggests that adhesion complexes containing E-cadherin form more aggregates that are reduced in size in the presence of olomoucine while cells containing N-cadherin increased in aggregate size (Figure 4L) with olomoucine. These results with the MDA-MB 231 cells therefore suggest that Cdk5 has differential effects on cell–cell junctions containing E-cadherin and N-cadherin, as suggested by experiments with N/N1003 lens cells and HCLE corneal epithelial cells (Figure 4A–K), and thus confirming the differential effects of Cdk5 on N-cadherin lens epithelial cells and E-cadherin-containing HCLE cells (Figure 4A–K) during cell–cell junction formation.

**Figure 5:**
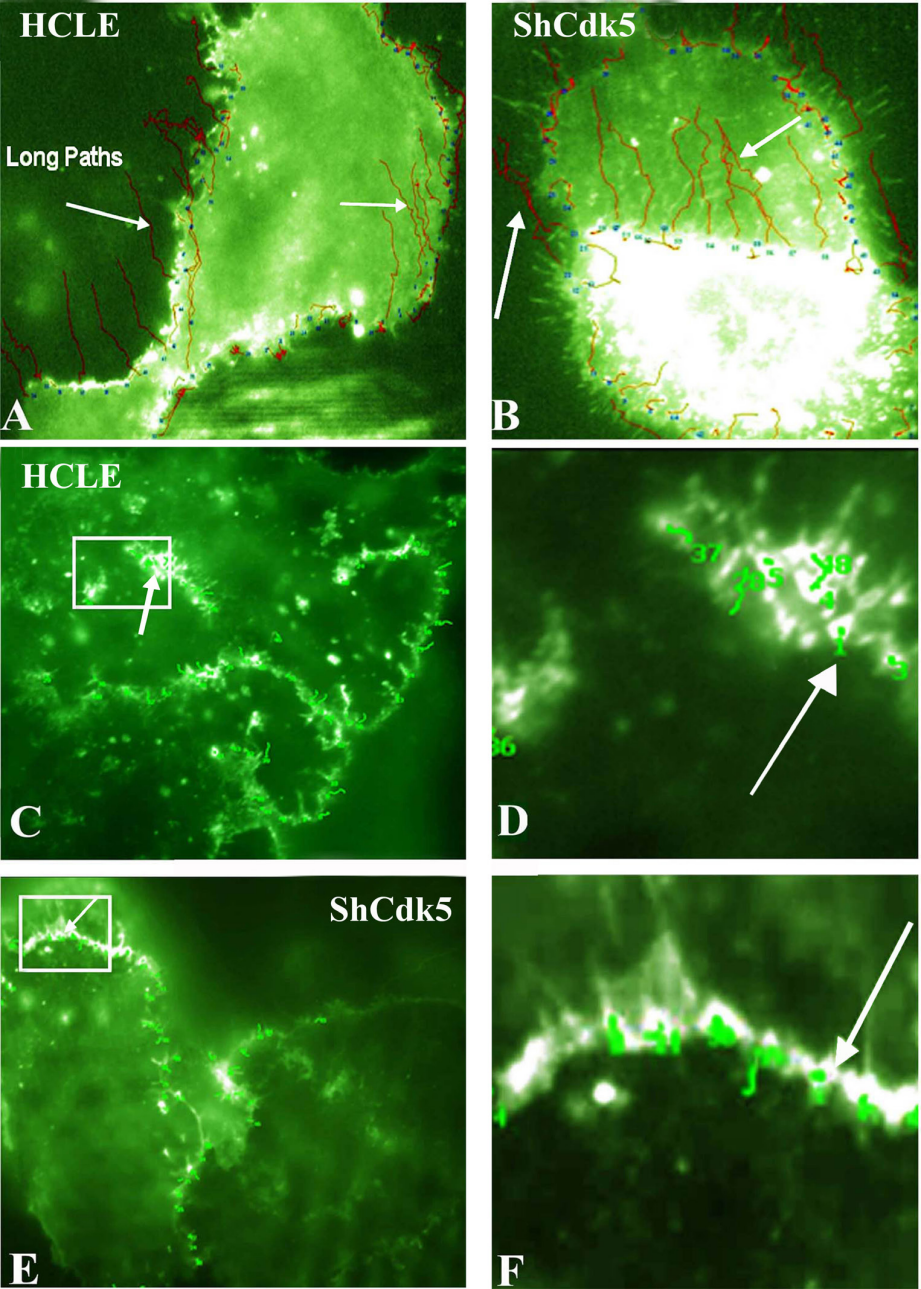
Total internal reflection fluorescence analysis for the E-cadherin internalization pathway in the human corneal limbal epithelial and ShCDK5 cells. Green fluorescent protein (GFP)-E-cadherin transfected (**A**–**B**) epithelial cells with and without olomoucine and ShCDK5 cultures were analyzed for E-cadherin particle movement for distance and tortuosity from the cell–cell borders at a constant time (**A**). The tortuosity or particle tracking of the E-cadherin-containing vesicles was recorded. The E-cadherin vesicles moved in long paths (arrows) from the cell borders to the interior, and such long paths were significantly greater in the ShCDK5 suggestive of internalization than the control human corneal limbal epithelial (HCLE) cells. Total internal reflection fluorescence (TIRF) analysis for the p120 (**C**–**H**) shows internalization pathway in the HCLE and ShCDK5. A GFP-p120 clone was transfected into the HCLE and ShCDK5 cells, and the vesicle tracking (arrows) for the tortuosity was measured. P120 vesicles remained in the cell–cell borders, and no long paths were internalized. In the HCLE and ShCDK5 cells, the p120 tortuosity paths were restricted within the cell–cell borders. Panel inserts in the HCLE cells transfected with p120 (**C**) and ShCdk5 transfected with p120 (**E**) are enlarged (zoom to a factor of 2) and shown in **D** and **F**, respectively, along with the tortuosity path for each junctional vesicle analyzed. The distance and tortuosity of paths by the E-cadherin- and p120-containing particles (n>100) were tracked at a constant time for all the particles analyzed using TIRF microscopy are described in Table 1. The E-cadherin-containing particles in the ShCdk5 cells moved fast, and these long, straight paths were internalized (**B**). The p120-containing particles in the ShCdk5 cells moved slowly and stayed near the cell–cell boundary (**C–F**). In the absence of CDK5, the E-cadherin and p120 junctional complex dissociate, promoting internalization and endocytosis of E-cadherin. Cdk5 stabilizes the junction by preventing E-cadherin-containing vesicles from being endocytosed. 100X images.

Interestingly, the loss of E-cadherin in the HCLE cells at the membrane led to the induction of N-cadherin. The dissociated E-cadherin from the junctional complex underwent internalization in the absence of Cdk5 leaving the p120 remaining at the membrane (Figure 5). Hence, we tested if the dissociated p120 at the membrane was bound to N-cadherin. Coimmuneprecipitation experiments revealed that inhibition of kinase activity was associated with increased binding of N-cadherin to p120. Cdk5 kinase activity is therefore required to prevent the dissociation and degradation of E-cadherin and the induction of N-cadherin (Figure 4M).

### Internalization of E-cadherin in the absence of cyclin-dependent kinase 5

We next used TIRF microscopy to investigate dynamic movements of E-cadherin and p120 in living cells. HCLE cells, olomoucine-treated cells, and ShCdk5 cells transfected with GFP-E-cadherin or GFP-p120 showed numerous, moving bright puncta at the cell–cell borders. Examining the velocity and tortuosity of the paths taken by a large number of such GFP-E-cadherin-labeled structures (n≥100 in three sets of experiments) demonstrated that the straight distance of particles traveled from the cell border to the interior with velocities ≤0.05 μm/sec in the HCLE cells was significantly (p≤0.05) reduced in contrast to the increase in the average distance of ShCdk5 (Figure 5) and olomoucine, suggesting internalization in the absence of Cdk5. However, highly tortuous particles that ended up remaining close to the membrane had a high tortuosity (up to 25.0) or a curved winding path. Such highly tortuous particles were found only in the p120 transfected HCLE and ShCdk5 cells.

TIRF analysis revealed two sub-populations of particles, namely, slow-moving particles (straight distance ≤1 μm, tortuosity ≤1.5) with velocities ≤0.05 μm/sec and slow-moving particles (straight distance ≥3 μm, tortuosity ≤1.5) and with velocities ranging from 0.09 to 0.6 μm/sec. The average length of all fast-moving particles that traversed from the cell–cell junctions to the interior was as long as 15–19 μm (Table 1). The slow-moving particles remained close to the cell–cell boundary while the fast-moving particles moved in long, straight paths and were directed away from the cell–cell border toward the cell interior. The E-cadherin particle rate of internalization from the cell border to its interior and the average length of particles was significantly increased (p≤0.005) in the absence of Cdk5 (Table 1). A subfraction of the fast-moving particles at a straight distance as long as 20 μm was specifically enhanced at a greater speed/velocity in the presence of olomoucine (36.633±4.08%) and in the ShCdk5 cell line (41.269±9.28%) when compared to the HCLE cells (26.90±3.60%; Figure 5; Table 1-column c (ii)). The proportion of E-cadherin particles internalized was increased in the absence of Cdk5. The length of the rapidly internalized particle was about 20 μm. The numbers/proportion of such particles that move from the cell border to the interior to a distance of 20 μm were increased only in the absence of CDK5 (Table 1).

**Table 1:** TIRF analysis of E-cadherin particle internalization.

**E-Cadherin** **Particle Movement**	**a** **Straight Distance**	**b** **Straight Distance** **Fast moving particles in Long straight Path**	**c** **% No. of fast moving internalized particles**	
			**(i)% Total of Fast moving Particles**	**(ii) %Subset of Fast moving Particles**
HCLE	7.292±0.54	15.368±1.03	29.211±6.70%	26.90±3.60%
ShCdk5	10.508±0.87*	19.541±1.02	39.105±4.34%*	41.269±9.28%
Olomoucine	10.74±0.53*	18.434±0.64	57.653±14.60%*	36.633±4.08%*

E-cadherin particle internalization from the cell border to the interior in HCLE was significantly reduced in contrast to the increased internalization of ShCdk5 and olomucine (*p≤0.05; a). The length of fast moving particles analyzed within the stipulated time for all the particles was 18um (b). Among the fast moving particles, a subset of particles ≥20um length internalized was significantly increased only in the ShCdk5 and olomucine (c). E-cadherin particle movement analyzed using TIRF therefore suggests that the particle rate of internalization and proportion of particles being internalized increased in the absence of Cdk5 Derivation: a. Average straight distance=Sum of straight distances/total number of straight distance. Internalized E- cadherin=particle movement b. Average Fast Moving Particles (distance ≥3μm,tortuosity ≤1.5)=sum of all the fast moving particles/tolat no. of fast moving particles. c. Proportion of fast moving internalized particles=No. of Fast moving Particles/total no of particles analyzedx100 (i) % Subset of fast moving internalized particles=No of fast moving particles at straight distance≥20µm tortuosity ≤1.5/ total no of fast moving particles x100 (ii). Results are expressed as average ± standard error mean.

In contrast to E-cadherin, movement of GFP-p120 particles was limited to the slowest velocities (mean of ≤0.5 um/sec), and mostly slow-moving particles remained at the cell boundary. This suggested that there were no fast-moving p120 particles. We therefore propose the p120 catenin is not internalized along with E-cadherin and may remain at cell–cell adhesion junctions without traversing to the cell interior even in the absence of Cdk5 (Figure 5). These findings imply that the inwardly directed E-cadherin is not bound to p120, suggesting that loss of Cdk5 promotes dissociation of E-cadherin from p120 at cell–cell junctions, followed by internalization of E-cadherin.

### Degradation of E-cadherin

Previous studies [29] have shown that E-cadherin is rapidly internalized and degraded when not complexed with p120. Therefore, we next tested whether inhibition of Cdk5 leads to degradation of E-cadherin. The HCLE cells were surface-biotinylated, blocked to prevent further reaction, and incubated with or without olomoucine (15 μM) for 4.5 h at 37 °C. The total and internalized biotinylated protein fractions were affinity purified on streptavidin-coupled beads, and the E-cadherin in the bound fraction was determined with immunoblotting. Inhibiting Cdk5 activity with olomoucine increased the loss of biotinylated E-cadherin approximately 1.7-fold (54±3% of the initial, biotinylated E-cadherin lost in 4.5 h in olomoucine-treated cells, compared to 32% ±3% in control cells). Thus, inhibiting Cdk5 activity destabilizes E-cadherin at the cell surface and increases the rate of E-cadherin degradation.

### Cyclin-dependent kinase 5 promotes cell–cell adhesion junction formation in human corneal limbal epithelial cells through G-proteins

**Figure 6:**
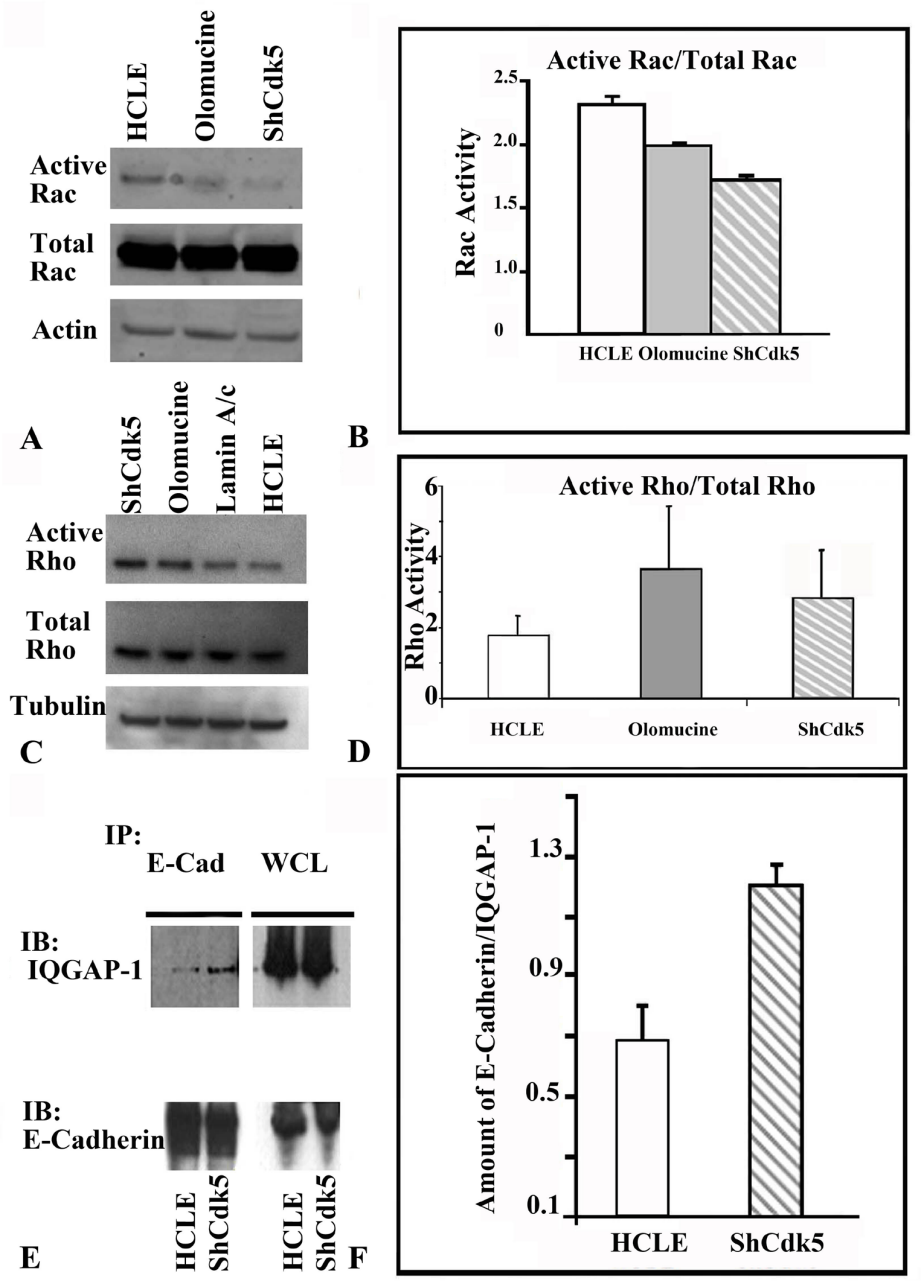
Rac activity is required for cell–cell adhesions in the human corneal limbal epithelial cells. The ShCDK5 human corneal limbal epithelial (HCLE) and olomoucine-treated cultures reduced Rac activity (**A**) leading to instability of the cell–cell adhesion junctions, while the HCLE cells have significantly higher Rac activity (**B**) Rho kinase activity was tested in the in ShCDK5 HCLE cells. **C**: Representative experiment showing a twofold increase in the Rho activity in confluent cultures of ShCDK5 and in olomoucine-treated cells when compared to the control Lamin A/C and HCLE. Significant (n=3; p≤0.05) increase in the Rho activity in the absence of Cdk5 with olomoucine (**D**) and decrease in Rac activity in the ShCdk5 and olomoucine, suggesting destabilization of cell–cell adhesions in the olomoucine and ShCDK5 cells, is represented in the graph (**B**, **D**). A cytoskeletal Rac modulator, IQGAP1, coimmunes precipitates with E-cadherin (**E**, **F**) in the ShCDK5 suggesting the internalization and degradation of E-cadherin. Stable cell–cell adhesions in HCLE cells are marked by significant reduction in the interaction of IQGAP1 with E-cadherin. (**E**, **F**). Note: Rho or Rac activity was measured as individual values normalized with the tubulin/actin and ratio of active Rho or active Rac against total Rho or total Rac, respectively. IB=immunoblot; IP=immunoprecipitation.

Since previous studies have shown that Rac activity is required to stabilize E-cadherin at cell–cell junctions [30], we next examined the effects of Cdk5 inhibition on Rac and its downstream effector, IQGAP1. IQGAP1 negatively regulates E-cadherin by interacting with β-catenin and displacing α-catenin from the adherens junctions. In its activated guanosine triphosphate (GTP)-bound form, Rac sequesters IQGAP1 and prevents its binding to β-catenin, thus stabilizing cadherin-mediated cell adhesion [9,31,32]. Immunoprecipitation results showed an increased association of IQGAP1 with E-cadherin (Figure 6) confirming a weak cell–cell junction that promotes degradation in the ShCdk5 cells. In addition, the loss of Cdk5 expression also inhibits the effects of active Rac (Figure 6). These results suggest that Cdk5 promotes the stability of E-cadherin in cell–cell adhesion junctions and protects E-cadherin from endocytosis and further degradation.

Rho is another candidate as a Rac1 regulator downstream of E-cadherin. Although several mechanisms have been proposed, it is well accepted that upon E-cadherin engagement, there is an elevation in Rac activity, which promotes the strength of cell–cell adhesion. Such a mechanism however, drastically suppresses Rho activity [33] leading to a concomitant increase in the activation of p190RhoGAP and thus suggesting the role of activating Rac by inhibiting Rho activity at the adhesion junctions [34]. We report that Rho activity in the presence of olomoucine and in ShCDK5 increased (Figure 6) while its negative regulator Rac was concomitantly decreased (Figure 6) in these weak adhesion junctions.

**Figure 7:**
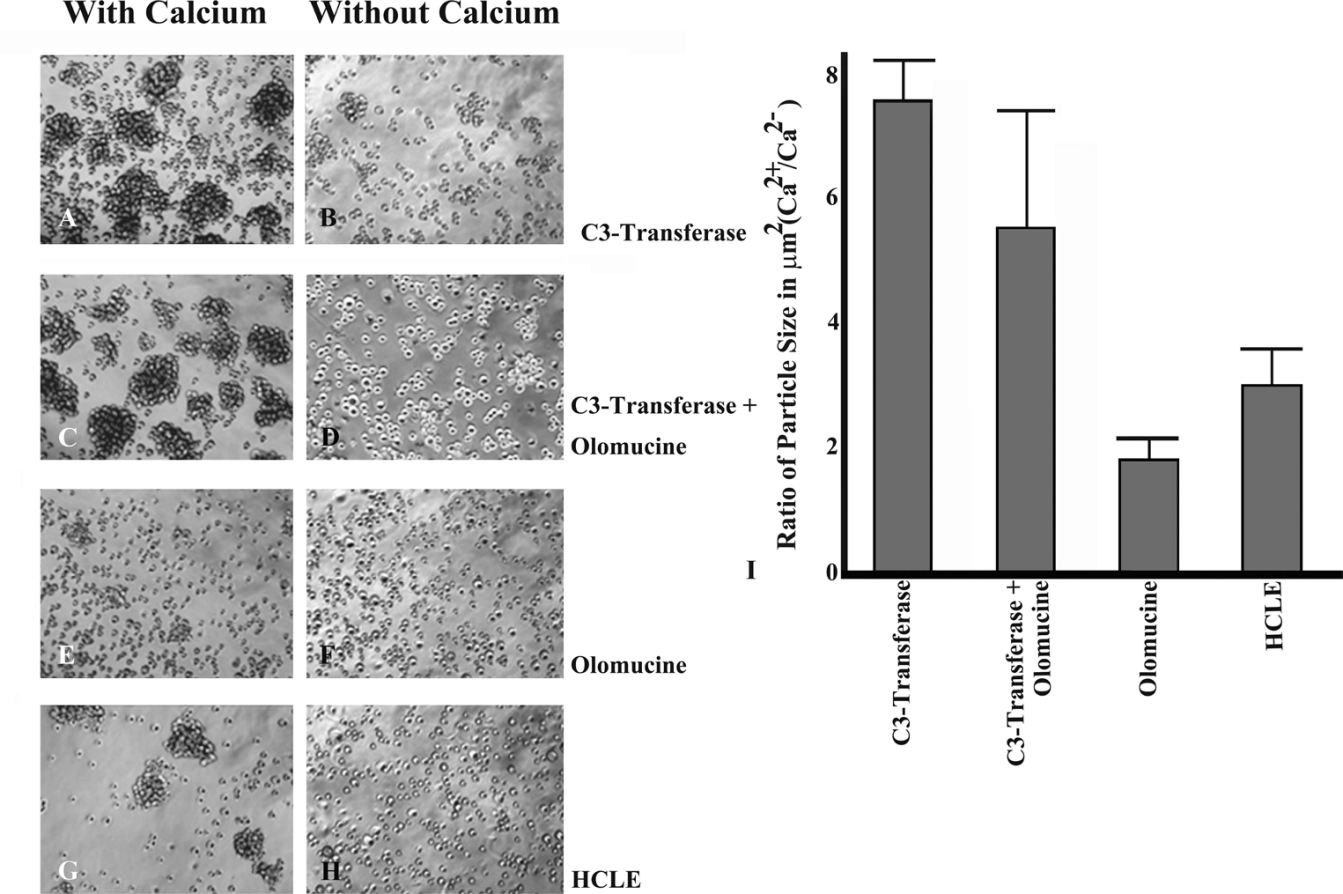
Rho activity mediates the downstream regulation of CDK5 in promoting cell–cell junction formation. Cell aggregates were measured with and without Ca^2+^ and defined as particle size in μm^2^ using NIH image J. In the presence of the C3 transferase, a Rho kinase inhibitor, the human corneal limbal epithelial (HCLE) cells form larger cell aggregates (**A**), and there is a reduction in the particle size in the presence of CDK5 and Rho kinase inhibitors suggesting the role of Cdk5 downstream signaling via the Rho kinase in the cell–cell junction formation (**C**). Olomoucine alone (**E**) reduces the formation of cell–cell aggregates than the control untreated (**G**) HCLE. Ratio of particle size=particle size (um) in the presence of calcium/ particle size (um) without calcium is represented in the graph (**I**). **A**–**H** are 20X images.

The role of Cdk5 in mediating the junctions via Rho kinase in the confluent HCLE cells was further tested. C3 transferase, a Rho kinase inhibitor, was used in the aggregation assay (Figure 7). Although aggregation increased in the presence of C3 transferase, dual inhibitors-olomoucine along with C3 transferase reduced the size of the aggregates. Therefore, the absence of Cdk5 kinase has an impact on Rho activity by disrupting the mature cell–cell adhesion junctions (Figure 6) and on forming new adhesion junctions (Figure 7) as opposed to positive effects of Rac activity requirement in junction formation [25] and cell junction stability (Figure 6). These results collectively suggest that Cdk5 plays a significant role in cell–cell adhesion formation, including junctional stability.

## Discussion

The mechanism of regulating stable cell–cell adhesion requires Cdk5. The present results indicate that Cdk5 activity is required for stable E-cadherin-dependent cell–cell junctions in corneal epithelial cells. This is consistent with previous findings [35-39] demonstrating that regulation of cell–cell and cell–matrix adhesion is coupled in migrating epithelial sheets and with our previous observation that inhibiting Cdk5 weakens corneal epithelial cell–cell junctions. We demonstrated that E-cadherin and the active form of Cdk5 phosphorylated at Y15 form a part of the same junctional complex. The shift in the localization of Y15 in the presence of olomoucine suggested the role of E-cadherin in maintaining a stable cell–cell junction. E-cadherin internalization has been associated with clathrin-mediated response [10] since the juxtamembrane region of the cadherin binds to other catenins, and the rate at which cadherin is recycled has been reported to depend on p120 catenin [40]. E-cadherin and p120 catenin tend to dislodge during the endocytotic response before the cadherin gets recycled to the cell membrane. TIRF analysis for particle tracking of adherens junction molecules suggests that olomoucine or ShCDK5 allows E-cadherin internalization. This process leaves the p120 at the cell membrane, which has shorter particle movement and tortuosity without being internalized along with E-cadherin and accounts for the turnover rate [30] of E-cadherin.

Regulators and downstream signaling targets of Cdk5 including activated GTP-bound Rho GTPases interact with specific effectors in modulating the E-cadherin-mediated adhesion. IQGAP1 may be involved in mediating the strength of cell–cell adhesions on the effect of Cdk5 on Rac. Rac1 effector IQGAP1 negatively regulates E-cadherin-mediated cell adhesion by interacting with β-catenin [9,32,40-44]. This is supported by the findings that Rac1 activity decreases after E-cadherin-mediated adhesion in the Cdk5-deficient adherent confluent epithelium. Thus, such a mechanism may be operating by Cdk5 at the cell–cell junctions in these cells. Moreover, recycling E-cadherin pool and internalization increases in the absence of stable cell–cell contacts in ShCdk5 supports the hypothesis that Cdk5 is required for maintaining adherent cell–cell adhesion junctions in the corneal epithelium. The binding of E-cadherin to IQGAP1 (Figure 6) and p120 [34,40] may also regulate the functional disassembly of cell–cell junctions via increased Rho activity (Figures 6, 7) [34,40]. The present study demonstrates that the increased Rac1 activity and the concomitant decrease in Rho activity and binding of IQGAP1 with E-cadherin downstream of CDK5 prevent the cadherin switch and protect the intact cadherin-catenin complex that would otherwise lead to epithelial mesenchymal changes. The internalized pool of surface biotinylated E-cadherin with olomoucine is a product of the gamma secretase complex that cleaves the intracellular domain of E-cadherin [45] leading to lysosomal degradation subjected to cytoskeletal changes by binding to the GTPases. Internalization or disassembly of E-cadherin promotes the induction of N-cadherin and binding to p120, a mechanism that upregulates Rho kinase activity by p120 [33,40,46] in corneal epithelial cells.

The lens epithelial cells containing N-cadherin and the corneal epithelial cells expressing E-cadherin are differentially regulated by CDK5 in the formation (Figure 4) and disassembly (Figure 7) of cell–cell adherens junctions. These results differ from previous findings of the role of Cdk5 in regulating cell–cell junctions in cells expressing primarily N-cadherin [22,47]. Our findings also provide evidence that the differing effects of Cdk5 are directly related to differences between E-cadherin and N-cadherin. The underlying mechanism therefore includes Rac/CDC42 downstream modulators of the IQGAP proteins that constitute the membrane cytoskeletal changes [48-50] and internalization of E-cadherin [51] to promote migration [13,22] via inhibiting de novo adherens, while Rho activity downstream of Cdk5 is implicated in the formation of cell–cell junctions. We have earlier shown that olomoucine reduced the size of corneal wounds without increasing the infiltrating inflammatory cells in the cornea and thus increased the rate of reepithelialization [2,13,20,22,24]. The present study therefore suggests the dualistic mechanism of regulation of Cdk5 that contributes to forming junctions, stabilizing the corneal epithelial cell adherens junctions, and targeting Cdk5 for potential therapeutic applications in cell–cell adhesion and migration.
